# Psychometric Evaluation of the ACHIEVE Assessment

**DOI:** 10.3389/fped.2020.00245

**Published:** 2020-05-29

**Authors:** Miriam Crowe, Donald Maciver, Robert Rush, Kirsty Forsyth

**Affiliations:** School of Health Sciences, Queen Margaret University, Queen Margaret University Drive, Edinburgh, United Kingdom

**Keywords:** social participation, psychometrics, child rehabilitation, disabled children, Inclusive education, Rasch analysis

## Abstract

**Objective:** There has been a significant change within clinical practice in childhood disability from “treating” at the level of body function to ecological approaches that address the child's involvement in everyday life. Clinical assessment, and robust tools to support this, are of key importance. The aim of this study was to assess the psychometric properties of the ACHIEVE Assessment in a clinical dataset. The ACHIEVE assessment is a parent and teacher report of participation in home, school and community settings, important contributory factors for participation, and environmental factors.

**Design:** ACHIEVE scores of children were collected from parents and teachers. The Rasch Rating Scale Model produced model estimates with WINSTEPS software.

**Setting:** Clinical rehabilitation settings in Scotland (United Kingdom).

**Subjects:** 401 parents and 335 teachers of 402 children participated resulting in a final sample of 736 responses. Children (78% male) were 4–17 years old (mean 7.91 years SD 2.61). Children had a range of disabilities including Developmental Coordination Disorder, Autism Spectrum Disorder, and Attention Deficit Hyperactivity Disorder.

**Results:** The study includes a large clinical sample of children with disabilities. The results demonstrate that the ACHIEVE Assessment can provide unidimensional measurements of children's participation and important contributory factors for participation. Differential item functioning analysis indicated majority of items were comparable between parent and teacher report.

**Conclusions:** The results confirm evidence of appropriate psychometric properties of the ACHIEVE Assessment. ACHIEVE is a comprehensive tool that enables identification of patterns and issues around participation for clinical and research purposes.

## Introduction

Participation is considered one of the most important aspects for personal, social, and academic development during childhood (1–3), supporting children's health, well-being and achievement (4–7). Key areas for assessment include home, school and community settings (8–11). A complex interaction of personal characteristics, skills, and motivation (9, 11, 12) as well as environmental factors (10, 13) influence participation. Recent developments (1) focus on a biopsychosocial conceptualization of child functioning, in particular not focusing solely on conditions (body functions and body structures), but instead aiming to assess and understand activities and participation in context (1). Such a focus is valuable tool for clinicians and researchers.

To support understanding of how to provide activity and participation-focused interventions for children with disabilities and their families, assessment should capture children's performance in variety of environmental and social conditions, including the home and school (14, 15). Gathering information from parents and teachers is therefore key. Previously, there were no instruments that provided teacher and parent reports (14). An instrument that supports collection of this data is desirable, to ensure family centered practice, and to enable understanding of children's participation in different contexts. Of the currently available assessments, few include multiple informants and/or provide an overarching portrait of the child's participation and key contributory factors for participation (14). The ACHIEVE Assessment was designed to meet these gaps, and was the focus of this work.

Traditionally, Classical Test Theory (CTT) has been used to support instrument development (16). More recently, Modern Measurement Theory (MMT) including the Rasch model are becoming the gold standard (16). The Rasch model infers linear, scaled measures from ordinal observations (16). Interpretations relating to unidimensionality, person and item validity, targeting and accuracy of a scale are produced (16, 17).

## Materials and Methods

### Study Purpose

The present study used a Rasch model approach to evaluate the ACHIEVE assessment in a clinical sample of children with disabilities.

### ACHIEVE Assessment

The ACHIEVE Assessment was designed to be used by clinicians to gather information from parents and teachers of children or young people aged 3–18 years. It was envisaged that clinicians would use the ACHIEVE Assessment in the assessment process, in order to make judgements about intervention, goal setting, necessity for further specialized assessment, or for post intervention evaluation. The ACHIEVE assessment consists of matched questionnaires (one to be completed by the parent/carer and one to be completed by the child's teacher).

### Items and Scoring

The assessment is supported by a 130 page manual (18). The assessment includes 54 items, and a 4-point frequency-based rating scale (1 = none of the time; 2 = some of the time; 3 = most of the time and; 4 = all of the time). The first set of items measure the frequency of children's activity participation in three settings (home activities 6 items, school activities 6 items, community activities 6 items). These items refer to the young person's engagement in those activities which they might be expected to perform. Parents and/or education professionals rate the frequency of engagement in each activity, therefore higher scores indicate greater frequency of participation. A further set of items aims to give an overview of aspects, which, collectively, define determinants, or contributory factors for participation: routines and roles (5 items), motivation (5 items), physical skills (5 items), social skills (5 items), and organizational skills (5 items). A further section has items focused on the environment (6 items). Parents and/or education professionals rate the frequency in which the young person displays behaviors relating to each area, and in the case of the environment, the frequency of the presence of supportive environmental aspects. Higher scores are indicative of the child or environment possessing favorable characteristics, likely to enhance, or not present barriers, to participation. There is an additional contributory factors section relating to “emotions and sensations” (5 items) that users are only directed to complete if a child is referred with Attention Deficit Hyperactivity Disorder (18). This is an optional section, where fewer responses would normally be expected, and is excluded from the current analysis.

Lower scores indicate areas to be explored in detail by the clinician to clarify need. As each child will ideally have two sets of scores (one from a parent and one from a teacher) results can be compared. A form is provided for the clinician to transcribe the scores from parents and teachers into one visual representation. This allows review and comparison of ratings. The clinician is enabled to develop an understanding of the child's needs and strengths across their participation and in relation to contributory factors for participation, supporting formulation of an intervention plan based on the child's specific needs. As the perspectives of the parent and the child's teacher are sought, use of ACHIEVE also facilitates a collaborative approach to assessment and goal setting.

### Conceptual Model Underpinning the ACHIEVE Assessment

The model underpinning the ACHIEVE Assessment is drawn from the Model of Human Occupation (MOHO) (19), a conceptual model from Occupational Therapy. The MOHO is a complex systems model, where people's activity and participation is defined as being driven by (1) motivation, habits, skills, and environment and (2) regular participation or performance of daily life activities (19). Participation is viewed as an emergent complex phenomenon, that occurs when people undertake, or perform, tasks related to clusters of life roles, which are always undertaken in the context of an environment, and in relation to people's motivations, habits, and skills (19). Hence, several aspects must be measured in tandem in order to gain a full overview. The MOHO conceptual framework and complex systems conceptualization is distinct from other models, for example the WHO-ICF (1). The MOHO provides an alternative theoretical perspective, meeting the calls for conceptual approaches beyond the ICF to guide measurement of activity and participation related constructs (15).

### Initial Development and Piloting

Development of item content drew from the MOHO conceptual framework, as well as previous research completed by academic development team (20–22). This previous work included consultation with wide range of individuals involving occupational therapists, physiotherapists, teachers, pediatricians, parents, psychologists, therapy assistants and managers on the key areas for assessment for children with disabilities (20–22). This work identified the need to gather information on children's participation within home, school and community settings and provided indications for likely content of items. In order to ensure further utility and clinical validity, a review of previously published assessments was also completed [see Supplementary File 1] where it was noted that no assessments of parent and teacher views were available. It was therefore seen as a unique strength of the ACHIEVE Assessment that it provides a means for gathering information about children's participation across multiple settings and from parent and teacher respondents. Items were finalized by a team consisting of occupational therapists, physiotherapists, and speech and language therapists, with input from international experts in MOHO and participation. A pilot version of the ACHIEVE Assessment was field tested in a children's occupational therapy service at a large teaching hospital in Scotland. A team of therapists used the assessment in practice over a 3 month period. Therapists provided their own feedback and also sourced comments from parents and teachers on the use of the tool. Key feedback was provided on clarity of instructions, item descriptions, and response format. This resulted in the version under assessment in the current study.

### The Current Study

#### Participants

Data were pre-intervention scores of 402 children with a range of disabilities including Developmental Coordination Disorder, Autism Spectrum Disorder, and Attention Deficit Hyperactivity Disorder who were referred for occupational therapy (OT) in Scotland (United Kingdom). Occupational therapy aims to enable people to participate in daily life to support their health, well-being and development (19). Typical areas of difficulty for children referred for OT include self-care (such as getting dressed), being productive (such as school e.g., handwriting) and leisure (such as playing with friends) (19). Typical conditions and disabilities seen by OTs include children with genetic and muscular skeletal disorders, sensory impairments, acquired brain injury, upper limb/digital deformity, general and emerging developmental delay as well as specific and pervasive developmental difficulties such as Developmental coordination disorder (DCD), Attention deficit hyperactivity disorder (ADHD) and Autism Spectrum Disorder (ASD).

Data were collected prospectively, based on clinicians using the ACHIEVE Assessment for the children on their caseload, over a 6 month period. Services were recruited through professional networks. Eleven services/teams (covering remote and rural areas as well as each major urban area in Scotland) were recruited. The participating services/teams were public and offered without charge, although a few private alternatives did exist, these were not commonly used or widely available in Scotland. The sample of services/teams was considered broadly representative of OT provision nationally.

#### Procedures

Following referral to OT, parents and teachers received the ACHIEVE assessments by post, returning the completed forms to the clinician. Written informed consent was then secured from parents, with information that they could leave the study, or not participate, at any time and for any reason, with no effect on their child's care. Forms were anonymized before returning to researchers. The study previously gained authorization from the National Research Ethics Service (NRES) and Queen Margaret University Ethics committee stating that full ethical approval was not required as anonymized clinical data were being collected. As the questionnaires were being used in practice within NHS Scotland services, an application was also made to the NHS National Caldicott Guardian scrutiny panel. This panel ensures that NHS data is safeguarded by reviewing applications to access such information and ensuring that principles of good practice, including strict adherence to confidentiality and data protections procedures. The application was approved in full and no further amendments requested. Researchers also made contact with relevant research and development (R&D) offices to gain local approvals.

### Statistics

In a Rasch context statistical analysis indicates how accurately or predictably data fit the model (23). A sample size of >250 provides >99% confidence in item calibrations within ± 0.5 logits (24). For the analysis, Winsteps (version 3.91.2) was used, with the Rating Scale Model and the JMLE (Joint Maximum Likelihood Estimation) method of estimation (25). SPSS (version 21.0.0) was used for data management and the sample demographics.

### Dimensionality

A Principal Component Analysis (PCA) of model residuals was used to identify non-Rasch dimensions beyond the expected latent construct (26). Comparison should be made between the variance explained and the unexplained variance in the 1^st^ contrast. An eigenvalue of at least 2–3 would be required to think of the variance as a “dimension” and not an idiosyncratic or poorly targeted item (27). Exploration of possible multidimensionality requires several associated assessments recommended by Linacre (26) (i) the off-dimension items should be identified and reviewed to check if they are substantively different, and if so, if they merit the construction of a separate test, and (ii) review of correlations between person measures on the item clusters using the following procedure. Items are anchored (fixed) at their difficulties from the main analysis. Then the items are segmented according to cluster, and each person is measured on each cluster. The person measures for each cluster of items are correlated with their measures from the other clusters. Each person measure for each cluster of items has a standard error which is removed to produce a disattenuated correlation. If the correlation approaches 1, then the measures from the clusters are statistically the same i.e., tapping into the same latent trait. Cut offs are that correlations <0.57 indicate multidimensionality, whilst correlations >0.71, >0.82, and >0.87 indicate increasing confidence that the items are measuring a unidimensional latent trait (26).

### Item Fit

Fit statistics provide a quantitative description of how well-individual items conform to the model of measurement (17). Fit statistics include the item locations (in logits), standard errors, residuals and fit to the model. Misfit implies that the item is not performing as intended and may not be measuring the intended construct. Individual item misfit was examined using the infit and outfit Mean-square statistics and the associated standardized fit, z-scores (23). The criteria for assessing item misfit was Mean-square >1.4 and a Z-value of >2.0 (17, 23). Infit is an information weighted statistic, outfit provides a score for the data at the upper and lower ranges, meaning that the statistic is sensitive to outliers (17, 23). Problematic outfit statistics are generally less of an overall threat to measurement than infit (17, 23). Items have high infit statistics when they do not measure the same dimension or construct as the other items in the set.

### Item Hierarchy

Rasch model analysis places persons and items on the same logit scale (log-odds units) (25). The location of items on the scale is important for content validity assessment. Review of ordering indicates whether items follow expected patterns, indicating construct validity (25).

### Targeting and Rating Scale

The targeting of the scale is assessed by comparing the correspondence between the mean person and mean item locations. A mean person location > mean item location reflects that the average item difficulty is below that of the sample. The rating scale average category measures and thresholds should increase according to guidelines (16).

### Differential Item Functioning

A Differential item Functioning (DIF) analysis is undertaken to assess if items perform differently for different groups (17), in this case the parent and teacher respondents. We identified values >0.64 *p* ≤ 0.01 as showing significant and meaningful DIF (17).

### Separation and Reliability

Separation and reliability for persons and items were calculated (16, 17). Separation gives an estimate of the spread of items or individuals along the continuum of ability and reflects the number of distinct strata into which the sample can be divided (16, 17). This is an indicator of instrument quality as it evaluates in terms of whether items can separate individuals into distinct levels of ability; the separation ratio may be transformed into strata index describing the number of significantly different levels. Reliability indicates how reproducible the person and item measure orders are. A person and item separation index >2 and reliability of >0.80 indicate good values (16, 17).

## Results

In total, 401 parents and 335 teachers of 402 children participated. One child's parent had consented, however did not complete the questionnaire and only a teacher form was available. Analyses were completed using the complete dataset (736 ACHIEVE forms, comprising 401 parents and 335 teachers). The mean age of children included in the study was 7.91 years (S.D. = 2.61). See Table 1 for the child sample descriptives. See Tables 2, 3 for a summary of Rasch indicators of measurement quality and detailed Rasch statistics across the ACHIEVE scales. See Table 4 for item estimates for teachers and parents and differential item functioning.

**Table 1:** Characteristics of children.

Children's reasons for referral^*^	***n* (%)**	**Age**	***n* (%)**	**Gender**	***n* (%)**
Fine motor skills	251 (62.4)	3y 6m−4y 5m	25 (6.2)	Female	81 (20.1)
Gross motor skills	218 (54.2)	4y 6m−5y 5m	35 (8.7)	Male	315 (78.4)
Handwriting	181 (45.0)	5y 6m−6y 5m	86 (21.4)	Missing	6 (1.5)
Organization	107 (26.6)	6y 6m−7y 5m	52 (12.9)		
DCD	89 (22.1)	7y 6m−8y 5m	42 (10.4)		
Dressing/washing	85 (21.1)	8y 6m−9y 5m	49 (12.2)		
Sensory processing	78 (19.4)	9y 6m−10y 5m	47 (11.7)		
ASD	52 (12.9)	10y 6m−11y 5m	15 (3.7)		
Academic/school	43 (10.7)	11y 6m−12y 5m	14 (3.5)		
Social interaction	36 (9.0)	12y 6m−13y 5m	12 (3.0)		
Feeding/drinking	35 (8.7)	13y 6m−14y 5m	5 (1.2)		
Behavioral or mental health	26 (6.5)	14y 6m−15y 5m	4 (1.0)		
Visual or auditory problems	23 (5.7)	15y 6m−16y 5m	2 (0.5)		
Developmental delay	21 (5.2)	16y 6m−17y 5m	2 (0.5)		
ADHD	20 (5.0)	Missing	12 (3.0)		
Learning disability	8 (2.0)				
Prematurity	7 (1.7)				
Seizures or epilepsy	3 (0.7)				
Cerebral palsy or ABI	2 (0.5)				
Equipment/assistive technology	1 (0.2)				
Missing	10 (2.5)				

* *Children may have multiple reasons for referral and allowing for multiple selections*.

*DCD, Developmental Coordination Disorder; ADHD, Attention Deficit Hyperactivity Disorder; ASD, Autism Spectrum Disorder; ABI, Acquired Brain Injury*.

**Table 2:** Summary table of instrument quality indicators.

**Criterion**	**Participation**	**Contributory factors**	**Physical skills**	**Environment**
***N*** **Items included in analysis**	18	20	5	6
**Person**				
Measure range, logits	−2.51 to 5.35	−6.48 to 6.08	−5.80 to 5.37	−1.72 to 3.61
Measure mean (SE), logits	0.84 (0.43)	0.14 (0.39)	0.29 (0.94)	1.37
Extreme persons, *N* (%)	10 (1.4)	3 (0.4)	40 (5.4)	65 (8.8)
Reliability	0.89	0.92	0.79	0.64
Separation	2.89	3.33	1.96	1.34
Strata^2^	4	4	2	2
**Item**				
Measure range, logits	−0.80 to 0.99	−1.09 to 1.34	−0.75 to 0.68	−1.08 to 1.77
Measure mean (SE), logits	0.00 (0.06)	0.00 (0.06)	0.00 (0.07)	0.00 (0.07)
Reliability	0.98	0.99	0.99	1
Separation	7.9	10.34	8.31	15.3
Strata^2^	10	14	11	20
Thresholds	None,−1.86,0.48,1.39	None, −2.13,0.43,1.70	None,−2.91,0.45,2.45	None,−1.68,0.2,1.48
Categories	−3.03,−0.80,0.99,2.71	−3.28,−0.92,1.10,2.97	−4.03,−1.25,1.47,3.64	−2.88,−0.83,0.91,2.75
**Dimensionality**				
Eigen value	2.94	2.82	1.87	1.73
% unexplained	8.3	7.1	16.4	14.2
% explained	49.1	49.9	56.3	50.6
**Disattenuated correlations**				
1–3	0.74	0.74	0.77	0.71
1–2	0.77	0.90	0.86	1.00
2–3	0.89	0.88	0.81	1.00

2 *Calculated as “H = (4G+1)/3”, where G = item/person separation (28)*.

**Table 3:** ACHIEVE assessment: measures, standard error, MnSq statistics, and differential item functioning (DIF) between parent and teacher responses.

	**Tag**	**Area**	**Detail**	**Measure**	**SE**	**Infit MnSq**	**Zstd**	**Outfit MnSq**	**Zstd**
Participation	8	School activities	Handwriting and shape making	0.99	0.06	1.19	3.49	1.20	3.52
	**6**	**Home/life activities**	**Transitions between activities**	0.89	0.05	0.96	−0.73	1.01	0.16
	4	Home/life activities	Cleans up after activity	0.37	0.05	0.93	−1.53	0.95	−0.97
	5	Home/life activities	Prepares self for school	0.37	0.05	0.96	−0.78	0.96	−0.74
	10	School activities	Engages in curriculum activities	0.34	0.05	0.91	−1.87	0.96	−0.63
	18	Community activities	Manages clothes after leisure activities	0.04	0.06	0.84	−3.24	0.80	−3.55
	2	Home/life activities	Manages clothing	0.02	0.05	0.85	−3.14	0.83	−3.11
	**11**	**School activities**	**Organizes self/cleans self**	0.00	0.05	0.99	−0.17	0.96	−0.61
	14	Community activities	Plays in organized group activities	0.00	0.05	0.88	−2.49	0.92	−1.44
	7	School activities	Effectively uses learning materials	−0.03	0.05	0.89	−2.37	0.93	−1.17
	9	School activities	Engages in sport activities	−0.03	0.05	0.94	−1.21	1.02	0.30
	15	Community activities	Participates in out of school clubs	−0.12	0.06	1.23	3.87	1.20	2.87
	13	Community activities	Rides a bike, scooter etc.	−0.14	0.06	1.56	8.97	1.52	7.17
	**3**	**Home/life activities**	**Manages snacks/lunch**	−0.28	0.06	0.98	−0.34	0.98	−0.30
	**17**	**Community activities**	**Participates in leisure activities**	−0.40	0.06	1.01	0.16	0.97	−0.37
	12	School activities	Dresses self after physical education	−0.52	0.06	0.84	−3.16	0.76	−3.98
	16	Community activities	Participates in social events	−0.69	0.06	0.97	−0.63	0.97	−0.45
	**1**	**Home/life activities**	**Cleans self after toilet**	−0.80	0.06	1.14	2.48	1.09	1.19
Contributory factors	36	Organization skills	Works out problems if stuck	1.34	0.06	0.84	−3.23	0.85	−2.98
	35	Organization skills	Maintains concentration	1.18	0.06	0.81	−3.71	0.86	−2.78
	37	Organization skills	Follows through instructions	0.62	0.06	0.64	−8.09	0.66	−7.45
	38	Organization skills	Completes activity steps in right order	0.46	0.06	0.75	−5.33	0.77	−4.88
	19	Routines/roles	Organizes routines	0.41	0.06	1.38	6.56	1.39	6.84
	34	Organization skills	Organizes and uses objects	0.33	0.06	0.82	−3.64	0.82	−3.75
	24	Motivation	Confident in abilities	0.30	0.06	0.89	−2.12	0.93	−1.37
	28	Motivation	Keeps trying despite challenges	0.07	0.06	1.10	1.86	1.12	2.24
	23	Routines/roles	Manages multiple responsibilities	0.02	0.06	0.81	−3.90	0.80	−4.08
	27	Motivation	Tells what wants to get better at	−0.04	0.06	1.29	5.22	1.28	4.85
	22	**Routines/Roles**	**Understands responsibilities**	−0.12	0.06	0.95	−0.91	0.93	−1.35
	26	Motivation	Satisfied with activity performance	−0.12	0.06	1.07	1.39	1.13	2.30
	33	Social skills	Asks for support needed	−0.12	0.06	1.23	4.21	1.21	3.75
	20	**Routines/Roles**	**Copes with changed routines**	−0.17	0.06	1.02	0.45	1.04	0.70
	32	Social skills	Understands others' feelings	−0.33	0.06	1.07	1.43	1.06	1.07
	29	Social skills	Plays/interacts well with others	−0.47	0.06	0.85	−3.19	0.85	−2.97
	25	Motivation	Enjoys daily activities	−0.57	0.06	0.93	−1.36	1.00	0.10
	31	Social skills	Speaks clearly with others	−0.73	0.06	1.30	5.56	1.29	4.80
	21	Routines/Roles	Copes with variety of activities	−0.96	0.06	0.88	−2.41	0.85	−2.77
	30	Social skills	Chatty/sociable and talks with friends	−1.09	0.06	1.17	3.30	1.17	2.69
Physical skills	39	Physical skills	Is not clumsy during activities	0.68	0.07	0.87	−2.57	0.86	−2.62
	42	Physical skills	Has adequate physical dexterity	0.66	0.07	0.85	−3.00	0.83	−3.35
	40	Physical skills	Does not lose balance during activities	−0.14	0.07	0.75	−5.27	0.74	−5.24
	41	Physical skills	Grips objects effectively during activities	−0.45	0.07	1.14	2.53	1.11	2.00
	43	Physical skills	Does not fatigue during activities	−0.75	0.07	1.38	6.55	1.40	6.40
Environment	**E6**	**Environment**	**Activities done usually**	1.77	0.06	1.17	3.16	1.15	2.77
	E1	Environment	Physical environment navigable	0.61	0.06	1.09	1.72	1.14	2.53
	E2	Environment	Environment has opportunities	−0.12	0.06	0.87	−2.37	0.82	−2.88
	E4	Environment	Adults available to support	−0.38	0.06	1.06	1.10	1.01	0.12
	E5	Environment	Environment facilitates activity	−0.81	0.07	0.87	−2.28	0.80	−2.59
	**E3**	**Environment**	**Child has access to equipment/objects**	−1.08	0.07	0.95	−0.79	0.92	−0.85

*Items in order of difficulty (most difficult at top)*.

*All items scale: (1 = none of the time; 2 = some of the time; 3 = most of the time and; 4 = all of the time). MnSq, Mean Square Fit Statistics; ZStd, Standardized Weighted/Unweighted Mean Square Fit Statistics. MnSq ideal is 1; Infit MnSq >1.4 with ZStd >2 indicates misfit*.

***BOLD items:** DIF contrast >0.64 with t-value >2.0 (p <0.01) between parent and teacher respondents*.

**Table 4:** Details of Differential Item Functioning (DIF) in the ACHIEVE Assessment items between teacher and parent responses.

	**Tag**	**Area**	**Detail**	**Respondent**	**Measure**	**SE**	**Respondent**	**Measure**	**SE**	**Contrast**	**SE**	***t***	***p***
Participation	**1**	**Home/life activities**	**Cleans self after toilet**	Teacher	−1.68	0.11	Parent	−0.27	0.07	−1.41	0.13	−11.00	0.00
	2	Home/life activities	Manages clothing	Teacher	−0.28	0.08	Parent	0.24	0.07	−0.52	0.11	−4.71	0.00
	**3**	**Home/life activities**	**Manages snacks/lunch**	Teacher	−0.88	0.09	Parent	0.12	0.07	−1.00	0.12	−8.60	0.00
	4	Home/life activities	Cleans up after activity	Teacher	0.34	0.08	Parent	0.40	0.07	−0.06	0.11	−0.53	0.60
	5	Home/life activities	Prepares self for school	Teacher	0.34	0.08	Parent	0.39	0.07	−0.06	0.11	−0.51	0.61
	**6**	**Home/life activities**	**Transitions between activities**	Teacher	0.50	0.08	Parent	1.21	0.07	−0.70	0.11	−6.44	0.00
	7	School activities	Effectively uses learning materials	Teacher	0.23	0.08	Parent	−0.23	0.07	0.46	0.11	4.24	0.00
	8	School activities	Handwriting and shape making	Teacher	1.33	0.08	Parent	0.72	0.07	0.61	0.11	5.48	0.00
	9	School activities	Engages in sport activities	Teacher	0.11	0.08	Parent	−0.14	0.07	0.26	0.11	2.34	0.02
	10	School activities	Engages in curriculum activities	Teacher	0.41	0.08	Parent	0.28	0.07	0.14	0.11	1.24	0.22
	**11**	**School activities**	**Organizes self/cleans self**	Teacher	0.53	0.08	Parent	−0.46	0.08	0.99	0.11	9.00	0.00
	12	School activities	Dresses self after physical education	Teacher	−0.56	0.09	Parent	−0.50	0.08	−0.07	0.12	−0.56	0.58
	13	Community activities	Rides a bike, scooter etc.	Teacher	−0.06	0.10	Parent	−0.19	0.07	0.13	0.12	1.08	0.28
	14	Community activities	Plays in organized group activities	Teacher	0.03	0.08	Parent	−0.03	0.07	0.06	0.11	0.56	0.58
	15	Community activities	Participates in out of school clubs	Teacher	0.10	0.10	Parent	−0.24	0.08	0.33	0.13	2.63	0.01
	16	Community activities	Participates in social events	Teacher	−0.58	0.09	Parent	−0.77	0.08	0.20	0.12	1.63	0.10
	**17**	**Community activities**	**Participates in leisure activities**	Teacher	0.07	0.09	Parent	−0.69	0.08	0.75	0.12	6.25	0.00
	18	Community activities	Manages clothes after leisure activities	Teacher	−0.08	0.10	Parent	0.10	0.07	−0.18	0.12	−1.50	0.13
Contributory factors	19	Routines/Roles	Organizes routines	Teacher	0.41	0.09	Parent	0.41	0.08	0.00	0.12	0.00	1.00
	**20**	**Routines/Roles**	**Copes with changed routines**	Teacher	−0.52	0.08	Parent	0.13	0.08	−0.65	0.11	−5.72	0.00
	21	Routines/Roles	Copes with variety of activities	Teacher	−1.21	0.09	Parent	−0.76	0.08	−0.45	0.12	−3.91	0.00
	**22**	**Routines/Roles**	**Understands responsibilities**	Teacher	−0.51	0.08	Parent	0.20	0.08	−0.72	0.11	−6.35	0.00
	23	Routines/Roles	Manages multiple responsibilities	Teacher	0.08	0.08	Parent	−0.03	0.08	0.11	0.11	0.93	0.35
	24	Motivation	Confident in abilities	Teacher	0.39	0.08	Parent	0.22	0.08	0.18	0.11	1.55	0.12
	25	Motivation	Enjoys daily activities	Teacher	−0.54	0.08	Parent	−0.61	0.08	0.07	0.11	0.63	0.53
	26	Motivation	Satisfied with activity performance	Teacher	−0.12	0.08	Parent	−0.12	0.08	0.00	0.11	0.00	1.00
	27	Motivation	Tells what wants to get better at	Teacher	0.23	0.08	Parent	−0.26	0.08	0.50	0.11	4.38	0.00
	28	Motivation	Keeps trying despite challenges	Teacher	0.07	0.08	Parent	0.07	0.08	0.00	0.11	0.00	1.00
	29	Social skills	Plays/interacts well with others	Teacher	−0.41	0.08	Parent	−0.52	0.07	0.11	0.11	0.99	0.32
	30	Social skills	Chatty/sociable and talks with friends	Teacher	−0.96	0.08	Parent	−1.21	0.08	0.25	0.11	2.16	0.03
	31	Social skills	Speaks clearly with others	Teacher	−0.58	0.08	Parent	−0.85	0.08	0.26	0.11	2.36	0.02
	32	Social skills	Understands others' feelings	Teacher	−0.08	0.08	Parent	−0.53	0.08	0.45	0.11	4.00	0.00
	33	Social skills	Asks for support needed	Teacher	0.14	0.08	Parent	−0.34	0.08	0.49	0.11	4.33	0.00
	34	Organization skills	Organizes and uses objects	Teacher	0.13	0.08	Parent	0.51	0.08	−0.39	0.11	−3.39	0.00
	35	Organization skills	Maintains concentration	Teacher	1.05	0.09	Parent	1.29	0.08	−0.24	0.12	−1.95	0.05
	36	Organization skills	Works out problems if stuck	Teacher	1.45	0.09	Parent	1.25	0.08	0.21	0.12	1.70	0.09
	37	Organization skills	Follows through instructions	Teacher	0.56	0.08	Parent	0.68	0.08	−0.12	0.12	−1.04	0.30
	38	Organization skills	Completes activity steps in right order	Teacher	0.43	0.08	Parent	0.49	0.08	−0.07	0.11	−0.58	0.56
Physical skills	39	Physical skills	Is not clumsy during activities	Teacher	0.52	0.10	Parent	0.82	0.09	−0.31	0.13	−2.30	0.02
	40	Physical skills	Does not lose balance during activities	Teacher	−0.23	0.10	Parent	−0.08	0.09	−0.15	0.13	−1.14	0.25
	41	Physical skills	Grips objects effectively during activities	Teacher	−0.36	0.10	Parent	−0.52	0.09	0.16	0.13	1.25	0.21
	42	Physical skills	Has adequate physical dexterity	Teacher	0.75	0.10	Parent	0.58	0.09	0.16	0.13	1.22	0.22
	43	Physical skills	Does not fatigue during activities	Teacher	−0.68	0.10	Parent	−0.81	0.09	0.13	0.13	0.96	0.34
Environment	E1	Environment	Physical environment navigable	Teacher	0.70	0.08	Parent	0.55	0.07	0.15	0.11	1.34	0.18
	E2	Environment	Environment has opportunities	Teacher	−0.36	0.10	Parent	0.03	0.08	−0.39	0.12	−3.17	0.00
	**E3**	**Environment**	**Child has access to equipment/objects**	Teacher	−0.56	0.10	Parent	−1.54	0.11	0.98	0.14	6.75	0.00
	E4	Environment	Adults available to support	Teacher	−0.10	0.09	Parent	−0.58	0.08	0.48	0.12	3.87	0.00
	E5	Environment	Environment facilitates activity	Teacher	−0.73	0.10	Parent	−0.87	0.09	0.14	0.14	1.01	0.31
	**E6**	**Environment**	**Activities done usually**	Teacher	1.24	0.09	Parent	2.17	0.08	−0.93	0.11	−8.12	0.00

***BOLD items:** DIF contrast >0.64 with t-value >2.0 (p <0.01) between parent and teacher respondents*.

### Participation

The participation scale included 18 items: home activities (6 items), school activities (6 items), and community activities (6 items). Inspection of dimensionality (Table 2) indicated that the model explained variance was 49% and the Eigenvalue of the first contrast (non-Rasch dimension) was 2.94, which accounted for 8% of the unexplained variation. This means that the non-Rasch dimension had the “strength” of about 3 items. Those items which may tap in to other constructs were identified and reviewed to check their relationships with the other clusters of items and if they merited splitting into a second test. On examining the disattenuated correlations between person measures on the item clusters, the items were highly correlated (0.74–0.89) indicating that the clusters of items were tapping into a similar latent trait.

In reviewing the performance of the scale (Table 2) the mean child measures were greater than that of the mean item measures; therefore there was an offset in the pitching of items to persons. Throughout, the observed category threshold measures monotonically increase in line with the category label, indicating that the categories capture increasing levels of the latent trait. Person and item reliabilities were both above the recommended minimum necessary for confidence in the measure calibrations. Separation statistics indicated that the items separated persons into at least 4 levels. Item difficulty estimates are presented in Table 3. The hardest items were 8 “Handwriting and shape making,” 6 “Transitions between activities,” 4 “Cleans up after activity,” and 5 “Prepares self for school”. The easiest items were 1 “Cleans self after toilet,” 16 “Participates in social events,” 12 “Dresses self after Physical Education” and 17 “Participates in leisure activities”. Item 13 “Rides a bike/scooter” showed misfit (MnSq 1.56, ZSTD 8.97), otherwise all items showed good fit. The decision was taken to proceed retaining that item, given one item misfitting is acceptable (25). Comparing the parent and teacher items, we found that there were five instances of DIF between parent and teacher ratings (Tables 3, 4).

### Contributory Factors

This analysis included the 25 contributory factors items: routines and roles (5 items), motivation (5 items), physical skills (5 items), social skills (5 items) and organizational skills (5 items). Initial analysis of dimensionality found the Eigenvalue of the first contrast was 3.31 with an associated unexplained variance of 7.4% (explained variance 45%). Those items which were potentially tapping into other constructs were identified and reviewed. The disattenuated correlations between person measures on the item clusters demonstrated a noticeable second dimension. On review, these items were tapping into physical skills. Therefore, those items pertaining to physical skills were removed and the analysis rerun with a new 20-item scale. With the new 20-item scale, characteristics improved. The resulting dimensionality investigation (Table 2) revealed evidence that was more supportive of a singular dimension, with first contrast Eigenvalue <3 and 7%, with variance explained close to 50%. The correlations between the groups of items in the 20-item scale were 0.74 to 0.90 providing evidence for unidimensionality.

In reviewing the performance of the 20-item scale (Table 2), the mean child measures were greater than that of the mean item measures; therefore there was an offset in the pitching of items to persons. The observed category threshold measures monotonically increased in line with the category label, indicating that the categories capture increasing levels of the latent trait. The reliability statistics for this set of items indicated strong item and person reliabilities. Separation statistics confirmed that the items separated persons into at least 4 levels. Item difficulty estimates are presented in Table 3. The hardest items were 36 “Works out problems if stuck,” 35 “Maintains concentration,” 37 “Follows through instructions” and 38 “Completes activities in the right order”. The easiest items were 30 “Chatty/sociable and talks with friends”, 21 “Copes with variety of activities,” 31 “Speaks clearly with others” and 25 “Enjoys daily activities.” We found that fit was good with all items falling into the desired range, there were two instances of DIF between parent and teacher versions (Tables 3, 4).

### Supplementary Analysis of Environment and Physical Skills Items

The remaining items were the six environment focused items, and the five physical skills items that had been removed from the contributory factors scale. These items were examined to consider their measurement properties as separate scales. First, the six environment items were reviewed (Table 2). The dimensionality analysis found the Eigenvalue for the first contrast as 1.73 with unexplained variance of 14% (explained variance 51%). Correlations between the item clusters were 0.71 to 1.00 indicating likely unidimensionality. The mean child measure was greater than that of the mean item measure; therefore there was an offset in the pitching of items to persons. The observed category threshold measures monotonically increased in line with the category label, indicating that the categories capture increasing levels of the latent trait. The person reliability was below (0.64) the desired level for this set of items. Separation statistics confirmed that the items separated persons into the minimum required of 2 levels. Item fit was good with all items falling into the desired range. We found that there were two instances of DIF between parent and teacher versions (Tables 3, 4).

For the five physical skills items, dimensionality analysis (Table 2) found the Eigenvalue for the first contrast was 1.87 with unexplained variance of 16% (explained variance 56%). Correlations between the item clusters were 0.77 to 0.86 indicating likely unidimensionality. With regards to measurement properties (Table 2) the mean child measure was greater than that of the mean item measure indicating a difficulty offset. The observed category threshold measures monotonically increased in line with the category label, indicating that the categories capture increasing levels of the latent trait. The person reliability was below (0.79) the desired level. Separation statistics confirmed that the items separated persons into the minimum required of 2 levels. Item fit was good with all items falling into the desired range and there were no instances of DIF between parent and teacher versions (Tables 3, 4).

## Discussion

Children's participation is an important outcome for pediatric healthcare providers (1, 2). The current study presents analyses of the ACHIEVE Assessment, a measure of children's participation and important contributory factors for participation. Analysis allowed in-depth investigation of the ACHIEVE Assessment. The focus of this study was on collecting clinical data that reflects the intended use of the ACHIEVE instrument. The study therefore included a nationwide clinical sample of children with disabilities ranging in age from 4 to 17 years. The nationwide sample reduces the potential of locality specific influences, and the large sample size enhances the precision of the Rasch model estimates. Our research adds to the growing body of work (29–31) using Rasch model analysis to examine children's participation measures.

Regarding the 18-item participation scale, the findings were largely encouraging. The low rate of misfitting items is a good indicator of acceptable measurement properties, and the dimensionality analysis suggested that the scale had a unidimensional structure. Item-difficulty hierarchies were found to be consistent with clinical expectations, providing further validity evidence. Reliability and separation statistics confirmed that the items were able to distinguish groups of children into four levels or strata. Regarding the 25-item contributory factors scale, no suitable model could be identified for the full item set. Dimensionality analysis indicated that physical skills items were tapping into a separate latent trait. These items were found to form an independent but still practically important dimension. However, although the physical skills items showed evidence of unidimensionality, they also showed poorer measurement properties than the main scale, and were only able to distinguish two levels or strata of performance. With the physical skills items removed, the new 20-item contributory factors scale, demonstrated good measurement properties. All items in this scale showed fit to the Rasch model, and evidence of unidimensionality. The 20-item scale was able to distinguish among groups of children, and differentiated four strata or levels of performance. Item difficulty hierarchies showed a sensible differentiation among easier and more difficult items.

The analysis treated the environment items as a separate dimension. Although this is a theoretically plausible decision as the WHO-ICF and MOHO differentiate between person and environment factors (1, 19) the fewer items and ease with which respondents endorsed the items reduces the precision with which it is possible to measure environments. One potential reason for the relative ease with which parents and teachers endorse the environment items is differences in respondent severity (16). Parents and teachers are rating their own environment which may make them reluctant to give lower scores or genuinely perceive the environment they provide to be supportive. Conversely, it may be that parents, who are actively participating in their child's care by, for example, completing an assessment, are already taking steps to provide supportive environments. For future researchers, adding more environment items to the ACHIEVE Assessment would enhance the conceptual relevance of the environment dimension, as well as increasing the precision of measurement

Some items demonstrated DIF between the parent and teacher versions. There are reasonable explanations for this. Each setting in which children engage exhibits genuine differences in the environmental demands placed on their participation (10, 32). Therefore, the varying ease with which each respondent endorses an item may reflect the demands of the setting. In addition, the parent-child relationship differs from the teacher-child relationship, thus altering the perspective of each respondent. Parents are more intimately involved with their children than teachers are and, particularly if they perceive their child to be struggling, may be more likely to see problems (33, 34).

Existing research highlights the value of including parent and teacher report when assessing children. Kersten et al. (2016), in their review of psychometric qualities of the Strengths and Difficulties Questionnaire (SDQ), suggest that the interest is not inter-rater reliability, but cross-informant consistency (35). When two respondents differ in their role or relationship to a child, it is not expected that they will rate them in the same way (35). Weak to moderate associations between parent and teacher report are seen in other research, for example in behavioral screening (35) and in research within children's mental health (36). Such associations are attributable to contextual influences, individual perceptions and genuine differences in children's behavior across environments (35, 36). However, there are significant benefits to multi-informant report in gathering information (7, 9, 14, 36). Gathering parent and teacher report means that an instrument is broadly applicable across different disabilities/issues. Whilst the family is a key influence on participation (37), there is particular value in gathering information from teachers (7). School participation is a growing area of research (38) and teacher-report instruments of school participation are novel. Overall, for the ACHIEVE assessment, results indicate that the parent and teacher versions are robust in their own right, but that some consideration should be made regarding direct comparisons.

### Recommendations

Excluding items which do not display favorable psychometric properties but which measure useful clinical components risks creating an overly narrow and homogeneous scale. The physical skills items and environment items were judged useful for clinicians, and as a common aspect of assessment, they should be retained in the tool. However, these scales are less likely to capture a comprehensive range of features, contain too few items, and demonstrate less than ideal measurement properties. Otherwise, the ACHIEVE scales may be seen as useful for clinicians in measuring participation and important contributory factors for participation. If total scores are desired, based on the analysis, it is recommended that these are taken by respondent (separating parent and teacher scores) for (1) participation (using parent or teacher ratings for home, school and community activities) and (2) contributory factors (using parent or teacher ratings for routines/roles, motivation, social skills, and organization skills). As noted, the evidence indicates that a summary score may also be taken for physical skills sub-section and for the environment sub-section, however though clinically useful, these should be treated with caution due to their poorer measurement qualities.

### Limitations

Generation of items was based on a rigorous development process; however, children with disabilities were not included. Further issues include limited demographic information collected about parents and teachers, as well as the lower number of older children included. Sampling was completed via non-random methods and because respondents could select multiple reasons for referral, these data are difficult to interpret. In many clinics, it would be unusual to see only two children with Cerebral Palsy (CP) over a period; however, the large group of children with “gross motor skills” or “fine motor skills” as the reason for referral are likely to include these children. In contrast, the larger proportion of children with DCD in the current study does reflect existing knowledge about typical occupational therapy caseloads. Lastly, of the frequently reported reasons for referral in this study, many reference functional impairments rather than diagnoses. This characteristic reflects the paradigm shift within practice where the focus is on the impact that disability has on a child's life, rather than presenting condition. However, the clinical nature of the sample is a strength, representing the population with which the instrument is intended for use. In addition, the ages of children and their reasons for referral broadly reflect those the instrument is targeted for. A final set of issues is that psychometrics only included Rasch-related analyses. Therefore, the test-retest reliability and criterion-related validity of the ACHIEVE scales are awaiting future scientific evaluation.

## Conclusions

The ACHIEVE Assessment has potential for use in practice and research. Future work should assess sensitivity to change over time to explore the possibility of using the instrument as an outcome measure.

## Data Availability Statement

The datasets generated for this study are available on request to the corresponding author.

## Ethics Statement

Ethical review and approval was not required for the study on human participants in accordance with the local legislation and institutional requirements. Written informed consent to participate in this study was provided by the participants' legal guardian/next of kin.

## Author Contributions

The study was initiated by DM, KF, and MC. DM, KF, RR, and MC wrote the paper. MC designed the study and collected the data. DM and MC monitored progress. Analysis was completed by MC and RR.

## Conflict of Interest

The authors declare that the research was conducted in the absence of any commercial or financial relationships that could be construed as a potential conflict of interest.

## Supplementary Material

Supplementary File 1 Assessment of previous tools.
