# Supplementary material for: Psychometric Evaluation of the ACHIEVE Assessment
Source: Front Pediatr. 2020 May 29;8:245. doi: 10.3389/fped.2020.00245 (PMC7272698; doi:10.3389/fped.2020.00245)
Supplement: Supplementary File 1 — Assessment of previous tools. [file Data_Sheet_1.docx]

Supplementary Material

**Additional file**

Prior to conducting the current study, the researchers carried out an extensive review of existing participation assessments. The work integrated information from existing assessment reviews and available psychometric studies. The review identified 34 assessments relevant to measuring children’s participation. Of these assessments, 9 are designed to capture children’s participation across home, school and community settings and have standalone articles with information pertaining to their validity and/or reliability (highlighted in bold in the appendix table). Although similar in their purpose, the assessments differ in their format, administration process and structure. Each assessment therefore has its own strengths in contributing to knowledge about children’s participation. However, a key limitation is that despite addressing participation across multiple settings, the assessments depend on single perspectives for their information. The SCOPE is the only assessment to provide a structure for incorporating teacher-report, however the time-intensive nature of interviews may prove restrictive in some clinical or research settings.

| Name | Review articles | Target group | Age Range | Respondent | Format | Setting | Items |
| --- | --- | --- | --- | --- | --- | --- | --- |
| APCP (Assessment of Preschool Children's Participation)^6^ | 4 | With and without disabilities | 2 - 6 years | Parent | Paper-based questionnaire, includes drawings of everyday activities | Home Nursery Child-care | 45 |
| APS (Assistance to Participate Scale)^7^ | 1, 2 | With disabilities | 5 - 18 years | Parent / caregiver | Paper-based questionnaire | Home Community | 8 |
| ASK-P (Activity Scale for Kids - Performance)^8^ | 1, 3 | Physical disabilities caused by neuromuscular disorders | 5 - 15 years | Child | Self-administered questionnaire, parents of children under 9 can read the questions | Home Community | 30 |
| CAMP (Caregiver Assessment of Movement Participation)^9^ |  | Motor problems | 5 years and up | Parent / caregiver | Questionnaire | Home | 35 |
| CAPE/PAC (Children's Assessment of Participation and Enjoyment)^10,11^ | 1 - 5 | With and without disabilities | 6 - 21 years | Child - with or without assistance | Self-administered or interviewer-administered questionnaire | Home. Community | 55 in each |
| CAQ (Community Activities Questionnaire)^12^ | 3 | With and without disabilities | 2 - 5 years | Parent / caregiver | Paper-based questionnaire | Community |  |
| **CASP (Child and Adolescent Scale of Participation) ^13^** | **1 - 5** | **Acquired brain injury** | **3 - 22 years** | **Parent or youth version** | **Paper-based questionnaire** | **Home School Community** | **20** |
| Child Engagement in Daily Life measure^14^ |  | Cerebral palsy | Up to 6 years | Parent | Questionnaire | Home Community | 18 |
| CHORES (Children Helping Out: Responsibilities, Expectations and Supports)^15^ | 1 - 3 | With and without disabilities | 6 - 11 years | Parent / caregiver | Paper-based questionnaire | Home | 33 |
| CLASS (Children's Leisure Assessment Scale)^16^ | 1 - 2 | With and without disabilities | 10 - 18 years | Child | Questionnaire | Home Community | 30, 5 open ended |
| **COSA (Child Occupational Self Assessment)^17^** |  | **With and without disabilities** | **8 - 13 years** | **Child** | **Paper checklist or card sort** | **Home**  **School Community** | **25** |
| **CPQ (Children Participation Questionnaire)^18^** | **1 - 3** | **With and without disabilities** | **4 - 6 years** | **Parent / caregiver** | **Paper-based questionnaire** | **Home**  **School Community** | **44** |
| FOCUS (Focus on the Outcomes of Communication Under Six)^19^ |  | Speech and language impairments | Up to 6 years | Parent | Questionnaire or interview | Home Community | 50 |
| FPQ (Frequency of Participation Questionnaire) | 3 | Cerebral palsy | 8 - 12 years | Parent / caregiver | Paper-based questionnaire | Home  School  Community |  |
| ICF-FAS (ICF Functional Assessment Scale)^20^ | 3 | Hearing impairment | 6 - 14 years | Parent / caregiver and/or teacher | NS |  |  |
| LAQ-CP (Lifestyle Assessment Questionnaire - Cerebral Palsy)^21^ | 3 | Cerebral palsy | 3 - 10 years | Parent / caregiver | Paper-based questionnaire | NS | 46 |
| LAQ-G (Lifestyle Assessment Questionnaire - Generic)^22^ | 3 | With and without disabilities | 5 - 7 years | Parent / caregiver | Paper-based questionnaire | Home Community | 53 |
| **LIFE-H (Assessment of Life Habits for Children)^23^** | **1 - 5** | **With disabilities** | **5 - 13 years** | **Parent / caregiver** | **Questionnaire or interview** | **Home**  **School**  **Community** | **197 (long) or 64 items (short)** |
| LPS-C (The Life Participation Scale for Attention-Deficit/Hyperactivity Disorder (ADHD)-Child Version)^24^ |  | ADHD | 6 - 17 years | Completed by interviewer asking parent /caregiver questions | Interview scale | Home Community | 24 |
| M2P1 (Mayo-Portland Participation Index Rating Form)- subscale from Mayo-Portland Adaptability Index^25^ | 5 | Acquired brain injury | Adults with guidelines for use with children | Child or parent, multiple respondents is preferable | Self-administered questionnaire | Home Community |  |
| National Survey of Schools and Environment^26^ | 3 | With disabilities | 5 - 18 years | School teacher | Paper-based questionnaire | School |  |
| **PACS (Paediatric Activity Sort Cards)** | **1 - 2** | **With and without disabilities** | **5 - 14 years** | **Child** | **Interview using sort cards** | **Home**  **School**  **Community** | **75** |
| PADL (Participation in Activities of Daily Living) | 1 - 2 | Chronic or acute health conditions | 6 - 18 years | Child | Interview | Home  School  Community | 12 |
| PCPQ (Pediatric Community Participation Questionnaire)^27^ | 1 - 3 | With disabilities | 8 - 20 years | Child | Questionnaire, can be administered by interview | Community | 19 |
| **PEM-CY (Participation and Environment Measure - Children and Youth)^28,29^** | **1, 3 - 4** | **With and without disabilities** | **5 - 17 years** | **Parent / caregiver** | **Web or paper-based questionnaire** | **Home**  **School**  **Community** | **25 participation items** |
| **PICO-Q (Participation in Childhood Occupations Questionnaire)^30^** | **1 - 2** | **With and without disabilities** | **6 - 10 years** | **Parent / caregiver** | **Paper-based questionnaire** | **Home**  **School**  **Community** | **22** |
| PIP (Pediatric Interest Profile) ALIP (Adolescent Leisure Interest Profile) | 3 | With and without disabilities | 12 - 21 years | Child | Self-administered questionnaire | Home Community | 83 |
| PIP (Pediatric Interest Profile) Kid Play Profile | 1, 3 | With and without disabilities | 6 - 9 years | Child | Paper-based questionnaire | Home Community | 50 |
| PIP (Pediatric Interest Profile) Preteen Play Profile | 1, 3 | With and without disabilities | 9 - 12 years | Child | Paper-based questionnaire | Home  Community | 59 |
| PLA (Participation in Life Activities Scale)^31^ |  | Children with asthma | 9 - 15 years | Child | Paper-based questionnaire | NS | Child selects 1-5 activities, asked 3 questions about each. |
| Preschool ACS (Preschool Activity Sort Cards) | 1 - 3 | With and without disabilities | 3 - 6 years | Parent / caregiver | Interview | Home Nursery Community |  |
| **QYPP (Questionnaire of Young People's Participation)^32^** | **4** | **With disabilities** | **14 - 21 years** | **Parent or young person** | **Self-administered questionnaire** | **Home**  **School Community** | **45** |
| **SCOPE (Short Child Occupational Profile)^33^** | **5** | **With disabilities** | **Birth to 21 years** | **Therapist** | **Information gathered from observation, discussion and records** | **Home**  **School Community** | **25** |
| SFA (School Function Assessment)^34-36^ | 1 - 5 | With and without disabilities | 5 - 12 years | School teacher and/or health professional | Observation and completion of test-booklet | School | 266 |

**Appendix references:**

1. Chien C, Rodger S, Copley J, Skorka K. Comparative Content Review of Children's Participation Measures Using the International Classification of Functioning, Disability and Health–Children and Youth. *Arch Phys Med Rehabil* 2014; 95: 141-152.

2. Chien C, Rodger S, Copley J, McLaren C. Measures of participation outcomes related to hand use for 2- to 12-year-old children with disabilities: a systematic review. *Child Care Health Dev* 2014; 40: 458-471.

3. Phillips R, L., Olds T, Boshoff K, Lane A, E. Measuring activity and participation in children and adolescents with disabilities: A literature review of available instruments. *Aust Occup Ther J* 2013; 60: 288-300.

4. Rainey L, van Nispen R, van dZ, van Rens G. Measurement properties of questionnaires assessing participation in children and adolescents with a disability: a systematic review. *Qual Life Res* 2014; 23: 2793-2808.

5. Ziviani J, Desha L, Feeney R, Boyd R. Measures of participation outcomes and environmental considerations for children with acquired brain injury: A systematic review. *Brain Imp* 2010; 11: 93-112.

6. Law M, King G, Petrenchik T, Kertoy M, Anaby D. The Assessment of Preschool Children's Participation: Internal Consistency and Construct Validity. *Phys Occup Ther Pediatr* 2012; 32: 272-287.

7. Bourke-Taylor H, Pallant JF. The Assistance to Participate Scale to measure play and leisure support for children with developmental disability: Update following Rasch analysis. *Child: Care, Health and Development* 2013; 39: 544-551.

8. Bagley AM, Gorton GE, Bjornson K, Bevans K, Stout JL, Narayanan U, et al. Factor- and item-level analyses of the 38-item Activities Scale for Kids-performance. *Dev Med Child Neurol* 2011; 53: 161-166.

9. Tsang KL, Bond T, Lo SK. Psychometric properties of the caregiver assessment of movement participation scale for screening children with development coordination disorders. *INT J DISABIL DEV EDUC* 2010; 57: 383-402.

10. Imms C. Review of the children's assessment of participation and enjoyment and the preferences for activity of children. *Phys Occup Ther Pediatr* 2008; 28: 386-401.

11. King G, Law M, King S, et al. Children's Assessment of Participation & Enjoyment & Preferences for Activities of Children (CAPE/PAC). : Pearson Clinical Assessment; 2004.

12. Ehrmann LC, Aeschleman SR, Svanum S. Parental reports of community activity patterns: A comparison between young children with disabilities and their nondisabled peers. *Res Dev Disabil* 1995; 16: 331-343.

13. Bedell G. Further validation of the Child and Adolescent Scale of Participation (CASP). *DEV NEUROREHABIL* 2009; 12: 342-351.

14. Chiarello LA, Palisano RJ, McCoy SW, Bartlett DJ, Wood A, Chang H, et al. Child Engagement in Daily Life: a measure of participation for young children with cerebral palsy. *Disabil Rehabil* 2014; 36: 1804-1816.

15. Dunn L. Validation of the CHORES: a measure of school-aged children's participation in household tasks. *SCAND J OCCUP THER* 2004; 11: 179-190.

16. Rosenblum S, Sachs D, Schreuer N. Reliability and Validity of the Children's Leisure Assessment Scale. *Am J Occup Ther* 2010; 64: 633-641.

17. Keller J, Kielhofner G. Psychometric characteristics of the Child Occupational Self-Assessment (COSA), part two: refining the psychometric properties. *SCAND J OCCUP THER* 2005; 12: 147-158.

18. Rosenberg L, Jarus T, Bart O. Development and initial validation of the Children Participation Questionnaire (CPQ). *Disabil Rehabil* 2010; 32: 1633-1644.

19. Washington K, Thomas-Stonell N, Oddson B, McLeod S, Warr-Leeper G, Robertson B, et al. Construct validity of the FOCUS^©^ ( Focus on the Outcomes of Communication Under Six): a communicative participation outcome measure for preschool children. *Child Care Health Dev* 2013; 39: 481-489.

20. Mishra A, Rangasayee R. Development of ICF based measuring tool for inclusive education set ups. *ASIA PAC DISABIL REHABIL J* 2010; 21: 57-69.

21. Mackie PC, Jessen EC, Jarvis SN. The lifestyle assessment questionnaire: an instrument to measure the impact of disability on the lives of children with cerebral palsy and their families. *Child Care Health Dev* 1998; 24: 473-486.

22. Jessen EC, Colver AF, Mackie PC, Jarvis SN. Development and validation of a tool to measure the impact of childhood disabilities on the lives of children and their families. *Child: Care, Health and Development* 2003; 29: 24-34.

23. Noreau L, Lepage C, Boissiere L, Picard R, Fougeyrollas P, Mathieu J, et al. Measuring participation in children with disabilities using the assessment of life habits. *Dev Med Child Neurol* 2007; 49: 666-671.

24. Saylor K, Buermeyer C, Sutton V, Faries D, Khan S, Schuh K. The Life Participation Scale for Attention-Deficit/Hyperactivity Disorder-Child Version: Psychometric properties of an adaptive change instrument. *J Child Adolesc Psychopharmacol* 2007; 17: 831-841.

25. Malec JF. The Mayo-Portland Participation Index: a brief and psychometrically sound measure of brain injury outcome. *Arch Phys Med Rehabil* 2004; 85: 1989-1996.

26. Simeonsson RJ, Carlson D, Huntington GS, McMillen JS, Brent JL. Students with disabilities: a national survey of participation in school activities. *Disabil Rehabil* 2001; 23: 49-63.

27. Washington LA, Wilson S, Engel JM, Jensen MP. Development and preliminary evaluation of a pediatric measure of community integration: The Pediatric Community Participation Questionnaire (PCPQ). *Rehabilitation Psychology* 2007; 52: 241-245.

28. Coster W, Bedell G, Law M, Khetani MA, Teplicky R, Liljenquist K, et al. Psychometric evaluation of the Participation and Environment Measure for Children and Youth. *Dev Med Child Neurol* 2011; 53: 1030-1037.

29. Coster W, Law M, Bedell G, Khetani M, Cousins M, Teplicky R. Development of the participation and environment measure for children and youth: conceptual basis. *Disabil Rehabil* 2012; 34: 238-246.

30. Bar-Shalita T, Yochman A, Shapiro-Rihtman T, Vatine J, Parush S. The Participation in Childhood Occupations Questionnaire (PICO-Q): a pilot study. *Phys Occup Ther Pediatr* 2009; 29: 295-310.

31. Kintner EK, Sikorskii A. Reliability and construct validity of the Participation in Life Activities Scale for children and adolescents with asthma: an instrument evaluation study. *Health Qual Life Outcomes* 2008; 6: 43-43.

32. Tuffrey C, Bateman B, J., Colver A, C. The Questionnaire of Young People's Participation ( QYPP): a new measure of participation frequency for disabled young people. *Child Care Health Dev* 2013; 39: 500-511.

33. Bowyer P, Lee J, Kramer J, Taylor RR, Kielhofner G. Determining the clinical utility of the Short Child Occupational Profile (SCOPE). *British Journal of Occupational Therapy* 2012; 75: 19-28.

34. Hwang J, Davies PL. Rasch analysis of the school function assessment provides additional evidence for the internal validity of the activity performance scales. *American Journal of Occupational Therapy* 2009; 63: 369-373.

35. Davies PL, Soon PL, Young M, Clausen-Yamaki A. Validity and reliability of the School Function Assessment in elementary school students with disabilities. *Phys Occup Ther Pediatr* 2004; 24: 23-43.

36. Hwang J, Davies PL, Taylor MP, Gavin WJ. Validation of School Function Assessment with elementary school children. *OTJR OCCUP PARTICIPATION HEALTH* 2002; 22: 48-58.
